# Characterization of mammary-specific disruptions for *Tph1* and *Lrp5* during murine lactation

**DOI:** 10.1038/s41598-017-15508-0

**Published:** 2017-11-09

**Authors:** Samantha R. Weaver, Nicholas J. Jury, Karen A. Gregerson, Nelson D. Horseman, Laura L. Hernandez

**Affiliations:** 1 Department of Dairy Science, University of Wisconsin-Madison, Madison, WI United States of America; 2 Neuroscience Graduate Program, College of Medicine, University of Cincinnati, Cincinnati, OH United States of America; 3 Department of Molecular and Cellular Physiology, College of Medicine, University of Cincinnati, Cincinnati, OH United States of America

## Abstract

Serotonin is a homeostatic regulator of the mammary gland during lactation. The contribution of mammary-derived serotonin to circulating serum serotonin concentrations was previously unknown. We have developed mice with mammary-specific disruptions of tryptophan hydroxylase 1 (*Tph1*) or low-density lipoprotein receptor-related protein 5 (*Lrp5*) that are induced during late pregnancy and lactation via use of the whey acidic protein (*WAP*)-*Cre* cre-lox system. Our objective was to characterize dams with a lactation- and mammary-specific disruption of *Lrp5* (*WAP-Cre* × *Lrp5* ^FL/FL^) or *Tph1* (*WAP-Cre* × *Tph1* ^FL/FL^). Milk yield and pup weights were recorded throughout lactation. Dams were euthanized on d10 postpartum and mammary glands and duodenal tissue were harvested. *WAP-Cre* × *Lrp5* ^FL/FL^ dams had elevated serotonin concentrations in both the mammary gland and circulation compared to controls. In contrast, *WAP-Cre* × *Tph1* ^FL/FL^ dams had decreased mammary gland and serum serotonin concentrations compared to controls. Alveolar morphology, milk yield, and pup weights were similar. Mammary-derived serotonin makes a significant contribution to circulating serotonin concentrations during lactation, with no effect on milk yield or alveolar morphology. These transgenic models can and should be confidently used in future lactation studies to further elucidate the contribution of serotonin to the maintenance of lactation.

## Introduction

Serotonin is an ancient molecule with conserved roles in cell signaling across the last two billion years of evolution^1^. With essential roles in immunity, mood, and the maintenance of homeostasis across several organ systems, it is not surprising that the exploration of serotonergic biology is a burgeoning field of study. Serotonin is produced in a two-step process from the amino acid L-tryptophan. The enzyme tryptophan hydroxylase (TPH) converts L-tryptophan to 5-hydroxy-L-tryptophan (5-HTP), which is then decarboxylated by aromatic amino acid decarboxylase (AADC) to yield serotonin. There are two genes encoding isoforms of TPH: *Tph1* is most abundant in the periphery, while *Tph2* is primarily expressed in the central nervous system^2^. Because serotonin cannot cross the blood-brain barrier, these two major pools of serotonin are largely considered to be independent of one another in the mammalian body^3^. A useful tool in the elucidation of serotonergic biology is a transgenic mouse model with a global disruption of *Tph1* (*Tph1* KO). By utilizing the *Tph1* KO mouse model, serotonin has been implicated in a variety of physiological processes. These include, but are not limited to, embryogenesis, organogenesis, gastrointestinal motility and homeostasis, immunity, insulin release from pancreatic beta cells, bone biology, liver regeneration, and mood regulation (for a review of the *Tph1* KO mouse in research, see^4^). In 2004, *Tph1* expression was identified in the mammary gland of the mouse, and it was shown that peripheral serotonin is a homeostatic regulator of the mammary gland^5^.

Low-density lipoprotein receptor-related protein 5 (*Lrp5*) inhibits *Tph1* expression in the duodenum^6^. In the mammary gland, *Lrp5* signals in response to Wnt ligands^7^ and therefore represents an attractive target for breast cancer therapies^8^. Given that *Lrp5* actively signals in the mammary gland and is an established inhibitor of serotonin signaling, it can be utilized to inhibit *Tph1* activity in the mammary gland.

Serotonin has been identified as a key player in the maintenance of homeostasis in the lactating mammary gland. In an autocrine-paracrine fashion, serotonin is known to regulate tight junctions between mammary epithelial cells to control milk volume^9^ and β-casein expression^10^, specifically through the serotonin receptor 7^11^ in mouse and human epithelial cells. Serotonergic regulation appears to be conserved across species: the bovine mammary gland is known to express several serotonin receptor isoforms^12^ and the *Myotis velifer* bat mammary gland shows differential expression of serotonin and *Tph* ^13^. Serotonin has also been implicated in the regulation of a variety of broader physiological processes during lactation. For example, serotonin modulates calcium homeostasis in rodents^14,15^, and both late lactation^16^ and transition period^17^ in dairy cows. In addition, serotonin affects mammary gland function following administration of a high fat diet in various rodent models^18,19^, and breast-to-bone communication^20^. Recently, a relationship has been suggested between the serotonergic and circadian systems in regulating mammary gland development and function^21^.

With the development of a *Cre*-mediated gene disruption system in the mammary gland through use of the whey acidic protein (*WAP*) promoter, it became possible to examine the contribution of particular genes specifically during lactation^22^. To this end, our objective was to examine the mammary-specific contributions of Tph1 and low-density lipoprotein receptor-related protein 5 (Lrp5), an inhibitor of Tph1, on circulating serotonin concentrations, and milk production. We report that disruption of the *Tph1* gene in the mammary gland during lactation reduces circulating serotonin concentrations, but does not alter milk synthesis.

## Results

### Mammary-specific *Tph1* disruption during lactation reduces serotonin content in the mammary gland, but not the duodenum

**Figure 1:**
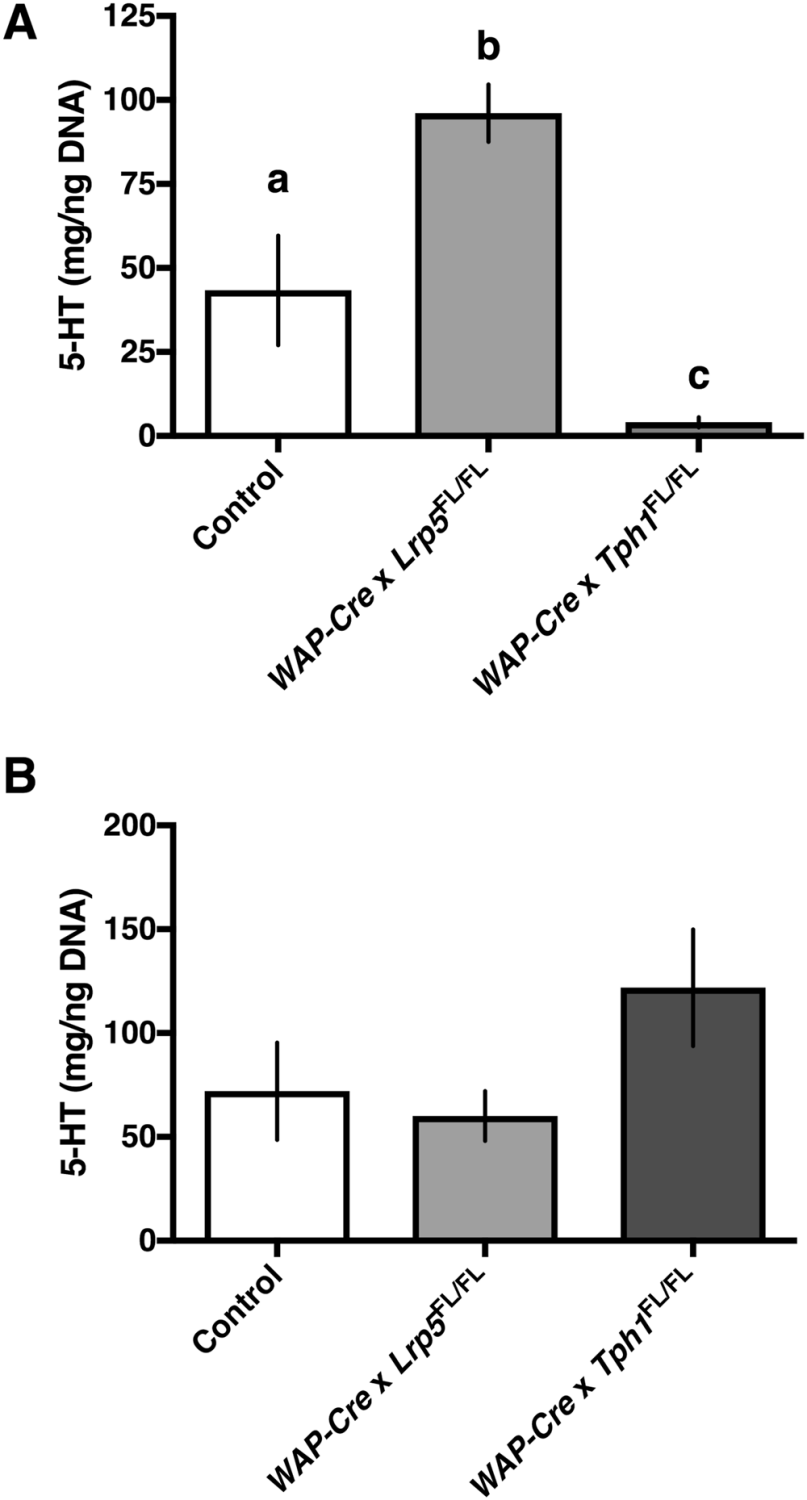
Mammary-specific *Tph1* disruption during lactation reduces serotonin content in the mammary gland, but not the duodenum. Control dams or dams with a mammary-specific disruption of *Tph1* or *Lrp5* during lactation were euthanized on d10 of their second lactation. Shown are the effects of genetic disruption on (**A**) mammary gland (*n* = 8 per treatment) and (**B**) duodenal (*n* = 5 for Control, *n* = 4 for *WAP-Cre* × *Lrp5* ^FL/FL^, *n* = 8 for *WAP-Cre* × *Tph1* ^FL/FL^) serotonin content. Values are means ± SEMs. Bars with same letter are not significantly different from one another. *WAP-Cre* × *Lrp5* ^FL/FL^, mammary- and lactation-specific disruption of *Lrp5*; *WAP-Cre* × *Tph1* ^FL/FL^, mammary- and lactation-specific disruption of *Tph1*.

Mammary gland and duodenal samples were collected on d10 postpartum and total serotonin content was measured. One-way ANOVA demonstrated that genotype had an effect on mammary gland serotonin content (Fig. 1A; *P* < 0.0001). Post hoc analysis demonstrated that *WAP-Cre* × *Lrp5* ^FL/FL^ dams had greater serotonin content in their mammary glands than CON dams (*P* < 0.01; 96.1 ± 8.6 vs. 43.4 ± 16.3 ng/mL for *WAP-Cre* × *Lrp5* ^FL/FL^ and CON dams, respectively). Additionally, *WAP-Cre* × *Tph1* ^FL/FL^ had very low mammary serotonin content (4.08 ± 1.5 ng/mL), compared to CON dams (*P* < 0.05) and *WAP-Cre* × *Lrp5* ^FL/FL^ dams (*P* < 0.0001). By contrast, there was no difference in serotonin content in the duodenum among the three treatments (Fig. 1B; *P* > 0.05).

### Serum serotonin mirrors mammary gland serotonin content, but serum tryptophan does not

**Figure 2:**
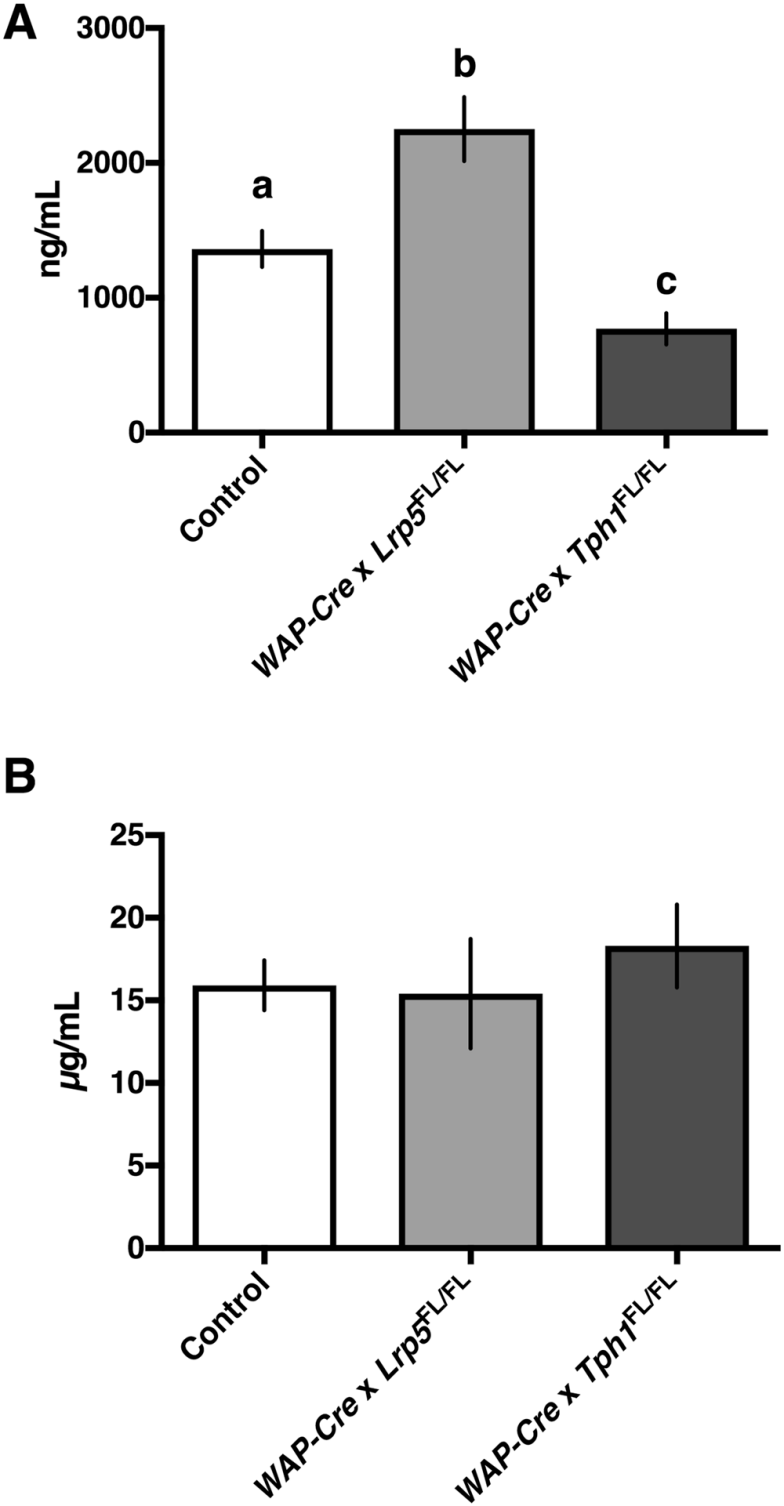
Dams with mammary gland disruption of *Tph1* or *Lrp5* during lactation have altered serum serotonin concentrations, but not altered tryptophan concentrations. Control dams or dams with a mammary-specific disruption of *Tph1* or *Lrp5* during lactation were euthanized on d10 of their second lactation. Shown are the effects of genetic disruption on serum (**A**) serotonin (*n* = 16 for Control, *n* = 10 for *WAP-Cre* × *Lrp5* ^FL/FL^, *n* = 10 for *WAP-Cre* × *Tph1* ^FL/FL^) and (**B**) tryptophan levels in the circulation (*n* = 15 for Control, *n* = 8 for *WAP-Cre* × *Lrp5* ^FL/FL^, *n* = 9 for *WAP-Cre* × *Tph1* ^FL/FL^). Values are means ± SEMs. Bars with same letter are not significantly different from one another. *WAP-Cre* × *Lrp5* ^FL/FL^, mammary- and lactation-specific disruption of *Lrp5*; *WAP-Cre* × *Tph1* ^FL/FL^, mammary- and lactation-specific disruption of *Tph1*.

The respective decrease and increase in mammary gland serotonin content in the *WAP-Cre* × *Tph1* ^FL/FL^ and *WAP-Cre* × *Lrp5* ^FL/FL^ dams, respectively, was reflected in their serum serotonin content. One-way ANOVA (*P* < 0.0001) followed by a Tukey’s multiple comparison post hoc analysis showed that *WAP-Cre* × *Lrp5* ^FL/FL^ dams had increased circulating serotonin (2252 ± 238 ng/mL) compared to either CON dams (1362 ± 133 ng/mL; *P* < 0.01) or *WAP-Cre* × *Tph1* ^FL/FL^ dams (770 ± 117 ng/mL; *P* < 0.0001). CON dams also had elevated circulating serotonin concentrations compared to *WAP-Cre* × *Tph1* ^FL/FL^ dams (*P* < 0.05; Fig. 2A). There was no difference between treatments with respect to circulating tryptophan concentrations (Fig. 2B; *P* > 0.05).

### Dams with a mammary-specific disruption of *Tph1* or *Lrp5* during lactation do not have altered alveolar morphology

**Figure 3:**
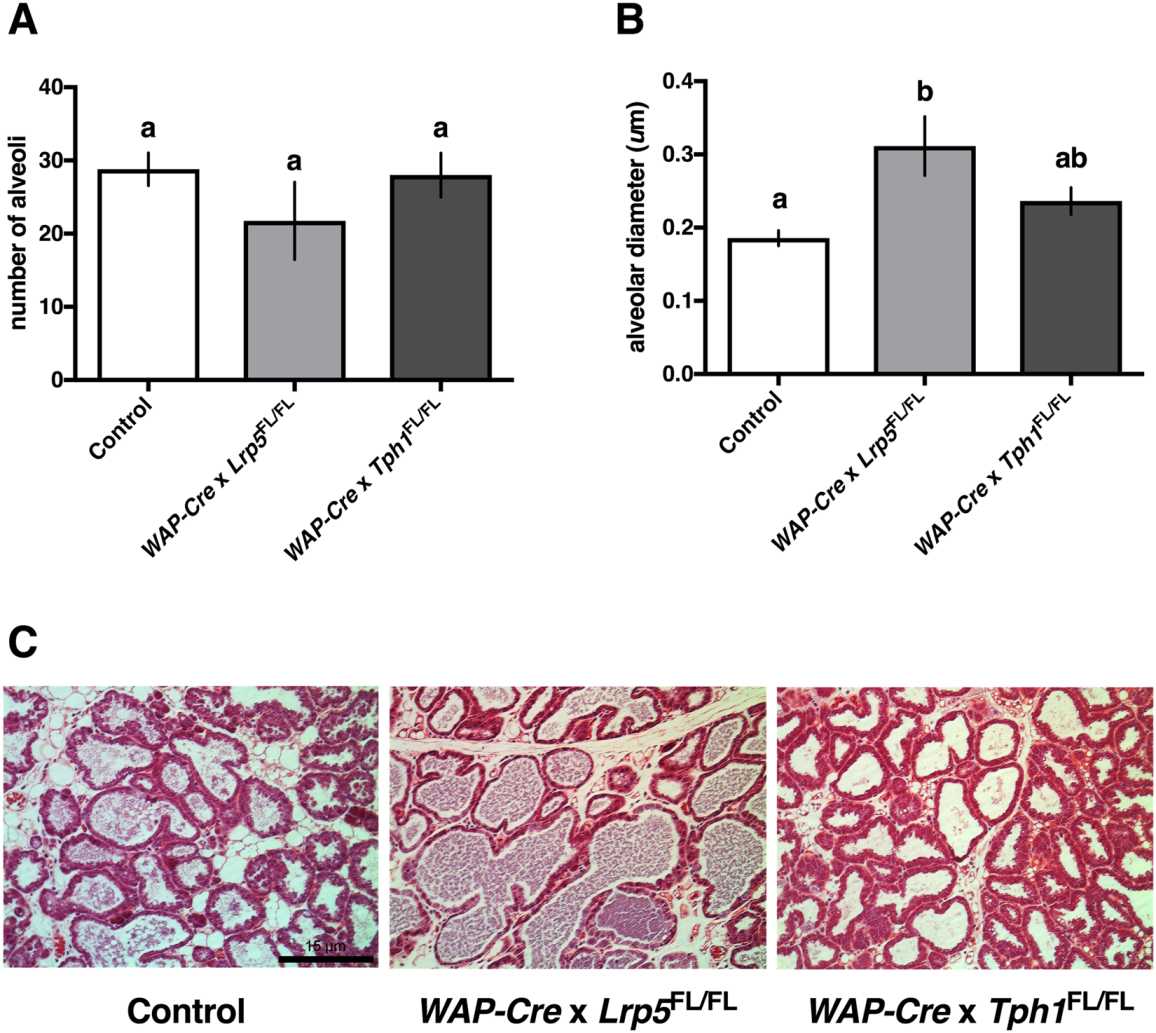
Dams with mammary gland disruption of *Lrp5* or *Tph1* during lactation do not have altered alveolar morphology. Control dams or dams with a mammary-specific disruption of *Tph1* or *Lrp5* during lactation were euthanized on d10 of their second lactation. Shown are the effects of genetic disruption on mammary gland (**A**) alveolar number (*n* = 5 for Control, *n* = 4 for *WAP-Cre* × *Lrp5* ^FL/FL^, *n* = 4 for *WAP-Cre* × *Tph1* ^FL/FL^) and (**B**) alveolar diameter (*n* = 5 for Control, *n* = 4 for *WAP-Cre* × *Lrp5* ^FL/FL^, *n* = 4 for *WAP-Cre* × *Tph1* ^FL/FL^). (**C**) Histological images stained by H&E and imaged at 20x magnification are depicted. The black scale bar on the Control image represents 15 μm. Values are means ± SEMs. Bars with same letter are not significantly different from one another. *WAP-Cre* × *Lrp5* ^FL/FL^, mammary- and lactation-specific disruption of *Lrp5*; *WAP-Cre* × *Tph1* ^FL/FL^, mammary- and lactation-specific disruption of *Tph1*.

Histological evaluation of the mammary glands harvested on d10 postpartum was performed to examine milk synthesis capacity. While *WAP-Cre* × *Lrp5* ^FL/FL^ have a numerically lower number of alveoli, there was no statistical difference detected between dams of different genotypes (Fig. 3A; *P* > 0.05). By contrast, a one-way ANOVA (*P* = 0.0005) followed by a Tukey’s multiple comparison post hoc analysis demonstrated that *WAP-Cre* × *Lrp5* ^FL/FL^ have alveoli with a larger diameter when compared with CON dams (*P* < 0.001; Fig. 3B). This is evident in the H&E images, where *WAP-Cre* × *Lrp5* ^FL/FL^ dams have visibly distended alveoli (Fig. 3C).

### Mammary-specific *Tph1* and *Lrp5* transgenic mice do not have altered milk yield or litter weights

**Figure 4:**
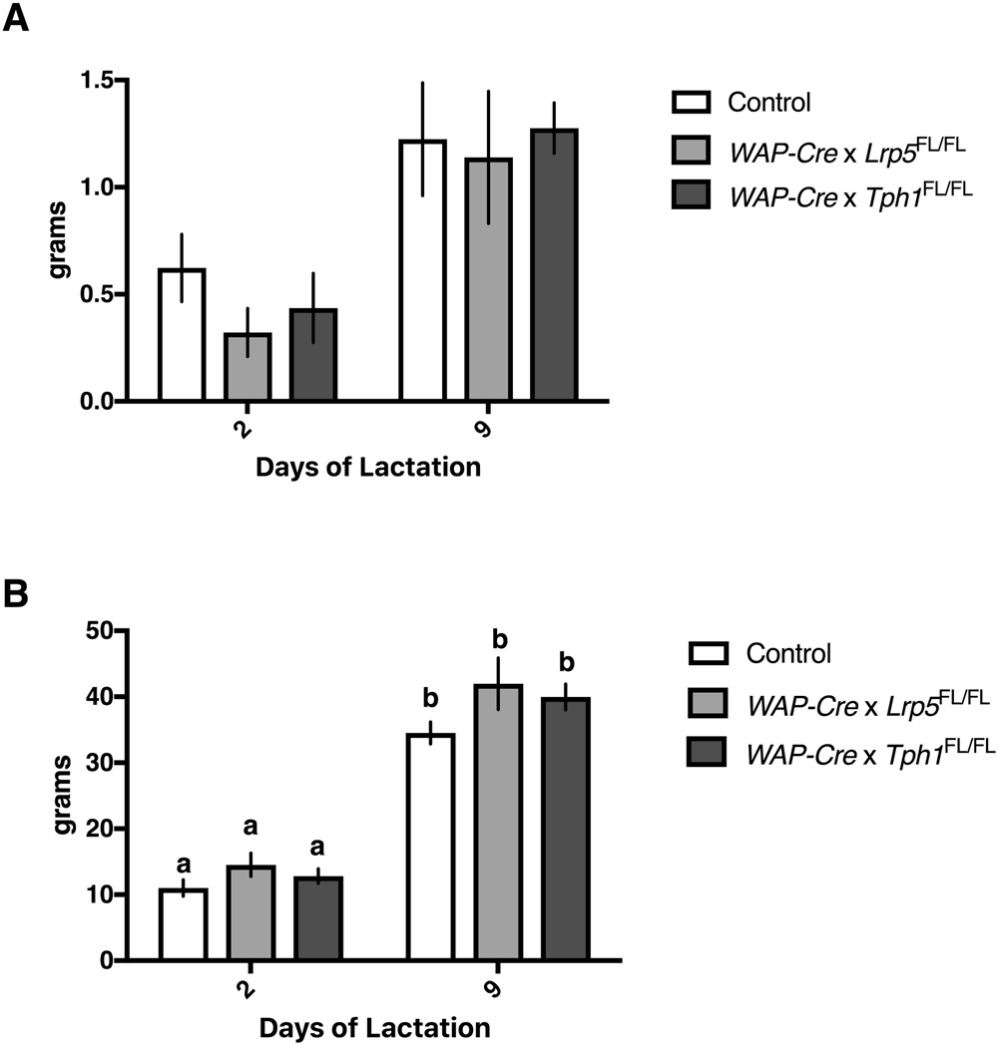
Mammary gland disruption of *Tph1* or *Lrp5* during lactation does not alter milk yields and litter weights. Control dams or dams with a mammary-specific disruption of *Tph1* or *Lrp5* during lactation were allowed to feed their pups throughout their second lactation. Shown are the effects of genetic disruption on (**A**) milk yield (*n* = 10 for Control, *n* = 10 for *WAP-Cre* × *Lrp5* ^FL/FL^, *n* = 8 for *WAP-Cre* × *Tph1* ^FL/FL^) and (**B**) litter weights (*n* = 10 for Control, *n* = 10 for *WAP-Cre* × *Lrp5* ^FL/FL^, *n* = 8 for *WAP-Cre* × *Tph1* ^FL/FL^) on d2 and d9 postpartum. Values are means ± SEMs. Bars with same letter are not significantly different from one another. *WAP-Cre* × *Lrp5* ^FL/FL^, mammary- and lactation-specific disruption of *Lrp5*; *WAP-Cre* × *Tph1* ^FL/FL^, mammary- and lactation-specific disruption of *Tph1*.

Although histological examination of the mammary glands did not demonstrate altered capacity to synthesize milk, it was still prudent to evaluate milk yield and pup growth. There was a main effect of time detected for milk yield, which increased from d2 to d9 postpartum across all dams (*P* < 0.0001). Two-way ANOVA also revealed that there was no effect due to genotype or interaction between genotype and time (*P* > 0.05; Fig. 4A). Similarly, there was a main effect of postpartum day on pup size (*P* < 0.0001), with all pups gaining weight from d2 to d9 postpartum, and no interaction between genotype and time (*P* > 0.05; Fig. 4B).

## Discussion

While serotonin is known to regulate mammary gland homeostasis during lactation in an autocrine-paracrine manner, it had not been possible to determine whether mammary-derived serotonin had effects outside the mammary glands^5,23^. Serum serotonin levels were found in a previous study to be elevated in lactating mice compared with nulliparous controls^24^. To determine the contribution of the mammary gland to circulating serotonin levels during lactation, we developed mice with mammary gland-specific disruptions for *Tph1* or *Lrp5*.

Dams with a mammary-specific disruption of *Tph1* during lactation have reduced serotonin content in their mammary glands, which is mirrored in the circulating serotonin concentration on d10 postpartum. By contrast, *WAP-Cre* × *Lrp5* ^FL/FL^ dams have increased mammary gland serotonin content and increased circulating serotonin concentrations. As such, our data show that during lactation the mammary gland is a major contributor to circulating serotonin stores. Because serotonin is derived from tryptophan, it was pertinent to examine tryptophan concentrations and elucidate whether there was any alteration in the serotonin synthesis pathway upstream of *Tph1*. As tryptophan concentrations were not significantly different between treatments, we are confident that it was the mammary-specific disruption of *Tph1* or *Lrp5* that altered circulating serotonin concentrations. In a non-lactating mammal, enterochromaffin cells and serotonergic neurons within the gastrointestinal tract (myenteric plexus) synthesize approximately 95% of serotonin in the periphery^25^. However, given that duodenal serotonin content was not altered in this experiment, we are confident that changes in circulating serotonin concentrations are a direct result of *Tph1* manipulation in the mammary gland. Future studies could include a Villin-Cre model with a gene disruption for Tph1 in the gut specifically in order to further elucidate the interaction of mammary- and gut-derived serotonergic pools^6^.

The finding that mammary serotonin contributes to circulating serotonin concentrations is important for the advancement of mammary gland biology. The mammary gland redirects various physiological events during lactation. For example, during lactation, the mammary gland sends signals that increase bone resorption and decrease kidney reabsorption of calcium while increasing gut calcium absorption^26^. Our findings demonstrate that whole-circulation serotonergic balance is also regulated by the mammary gland, redirecting physiological homeostasis to meet the needs of lactation. During lactation, the mammary gland appears to have compensatory mechanisms in place to sustain milk synthesis, demonstrating the importance of proper homeorhesis during this crucial time in the mother’s life^27^.

Although all of the dams in this experiment were able to sustain lactation and support their litters, *WAP-Cre* × *Lrp5* ^FL/FL^ had slightly fewer, but larger alveoli. Expression of *Tph1* is inhibited by co-expression of *Lrp5*, explaining why disruption of *Lrp5* lead to increased serotonin content in the mammary gland^6^. The interaction of Lrp5 or Lrp6 and a Frizzled receptor plus a Wnt ligand results in the recruitment of axin from the beta-catenin destruction complex, stabilization of beta-catenin, and subsequent transactivation of specific target genes. This canonical Wnt signaling is difficult to study in the adult stages of mammary gland development, as inhibition of Wnt signaling prevents gland formation^28^. As such, we cannot rule out the possibility that perturbation of the Wnt pathway itself was mediating alveolar diameter and number. Importantly, mice with a disruption for *Lrp5* have previously been shown to lactate normally, with normal mammary ductal trees^29^. Given these findings, it is likely the rise in serotonin content as a result of *Lrp5* disruption that provokes any mammary phenotype, and not Wnt signaling alone.

Serotonin regulates milk synthesis in a time-, environment- and dose-dependent manner. At high concentrations, serotonin can reduce milk yield by stimulating mammary gland involution^9^. Mammary alveoli become distended in response to milk stasis, ultimately causing the cells to shed and die. In serotonin receptor 7 knockout mice, the mammary epithelium retains a columnar shape, with reduced cell shedding, suggesting that serotonin mediates alveolar distension^11^. Although there were no differences in milk synthesis, despite *WAP-Cre* × *Lrp5* ^FL/FL^ having fewer and larger alveoli, it would be interesting to evaluate the involution process more rigorously in our mouse models, in order to establish the relationship between alveolar distension and involution in response to autocrine-paracrine serotonin.

Despite slight histological differences, data from the present study demonstrate that the ability to produce milk and support pups was not affected by mammary-specific manipulation of the serotonergic system. Elevated concentrations of serotonin stimulate involution^9^ and disruption of *Tph1* rescued milk synthesis in the HFD-fed mammary gland^19^. As such, while *elevated* serotonin activity may negatively affect milk yield, our data suggests that the mammary gland has compensatory mechanisms to assure proper milk synthesis in the *absence* of serotonin. A cross-fostering experiment with exploration of the effects of litter size and lactation persistency would be necessary to confirm this hypothesis. While it is standard practice to standardize litter size in lactation studies to control for metabolic output of the dam^30,31^ it would be relevant to examine the effects of larger litters putting higher demand on the dam and over a longer course of time, given serotonin’s role in lactation persistency^9^. Additionally, more detailed studies examining the effect of mammary-specific *Tph1* gene disruption on involution and the cellular architecture of the mammary epithelial cell may help elucidate the effect of serotonin on milk synthesis capacity in the future. Finally, peripheral serotonin is known to regulate feed intake and energy metabolism^24,32,33^, which may subsequently affect milk yield and pup growth. Maternal feed intake and body weight were not examined in this study, but further studies should be directed at the role of mammary-derived serotonin in regulating feed intake and subsequent milk yield during pregnancy and lactation.

The mammary-specific contribution to serotonergic homeostasis during lactation was previously unknown. Here, we report that mammary-derived serotonin makes a significant contribution to circulating serotonin concentrations during lactation. We also show that mammary-derived serotonin is not rate-limiting for lactation, as *WAP-Cre* × *Tph1* ^FL/FL^ and *WAP-Cre* × *Lrp5* ^FL/FL^ dams were able to successfully support pups and have normal alveolar morphology. Future studies should include the use of global *Tph1* knockout mice as well as global *Tph2* knockout mice in order to tease out the intricacies of central-to-peripheral serotonin communication with respect to mammary biology and maternal behavior. We conclude that use of these transgenic models can be confidently pursued in future research studies during lactation. Given that serotonin is a key regulator of mammary gland development and maintenance of lactation, these findings are broadly impactful to the field of reproductive biology.

## Methods

### Animal Handling

All procedures were reviewed and approved by the University of Cincinnati Institutional Animal Care and Use Committee. All experiments were performed in accordance with relevant guidelines and regulations. The experiments utilized two transgenic mouse lines where *Tph1* expression was either disrupted, or was overexpressed via disruption of *Lrp5*, a *Tph1* inhibitor^6^. Mice on the C57/BL6J background in which either the *Tph1* or *Lrp5* gene was flanked with two loxP (FL/FL) sites globally were obtained from the laboratory of Dr. Gerard Karsenty at Columbia University. Transgenic mice homozygous for the floxed genes were crossed with Agouti mice on an FVB background that express *Cre* recombinase under the control of the *WAP* promoter, which were a generous gift from Dr. Susan Waltz at the University of Cincinnati College of Medicine^22,34^. During late pregnancy (approximately embryonic day 18 in mice) and lactation, WAP production drives the expression of *Cre* within mammary epithelial cells, causing gene disruption of either *Tph1* or *Lrp5* selectively in the mammary gland. Previous work has demonstrated that a global disruption of *Tph1* ^2,5^. and a global disruption of *Lrp5* ^6^ does not inhibit lactation. Female mice (age 3 to 6 months) homozygous for loxP sites (FL/FL) flanking the *Lrp5* or *Tph1* gene were used as the control group (CON; *n* = 16). No physiological differences were observed between mice in which the *Tph1* or *Lrp5* FL/FL was not induced by a *Cre* recombinase; therefore, the two genotypes were considered to be phenotypically equivalent. An unpaired t-test showed that serum serotonin on d10 postpartum was 1529 ± 217 vs. 1196 ± 145 ng/mL for *Lrp* FL/FL and *Tph1* FL/FL mice, respectively (*P* = 0.22). The two transgenic mouse lines characterized were those mice with either the *Lrp5* FL/FL (*WAP-Cre* × *Lrp5* ^FL/FL^; *n* = 10) or *Tph1* FL/FL (*WAP-Cre* × *Tph1* ^FL/FL^; *n* = 10) and the inducible *Cre*-driven WAP promoter.

Three females per treatment were harem mated with a stud male. Previous studies showed that there were still intact homozygous loxP alleles present within mammary epithelial cells after one pregnancy and lactation cycle^35^, and that WAP itself is not fully induced through a single pregnancy^35^. Consequently, mice were first allowed to complete a full cycle of pregnancy and lactation, and were then allowed to mate a second time. Upon conformation of the second pregnancy, females were separated and individually housed on a 12 h light:dark cycle with water and standard lab chow available *ad libitum*. Litters were standardized to six pups on d2 postpartum following the second pregnancy and allowed to lactate through d10 of lactation. The dams were then euthanized, and serum, duodenum, and mammary gland samples were acquired.

### Milk Yield and Pup Growth

On d2 and d9 of the second pregnancy, milk yield was evaluated by the weigh-suckle-weigh (WSW) method. Briefly, this method consists of removing the pups from the dam for 4 hours, weighing the pups, replacing them with the dam, and weighing them again after pups are allowed to suckle for 45 minutes^18^. Daily litter weights to evaluate pup growth on d2 and d9 postpartum were considered the first weight collected before the 45 minutes of suckling.

### Blood and Tissue Collection

Twenty minutes after the FST on d10 of the second lactation, dams were euthanized and trunk blood was collected. The left 4^th^ mammary gland and the duodenum were removed for evaluation of serotonin content, while the right 4^th^ mammary gland was removed for histological evaluation. Mammary and duodenal tissues used for determination of serotonin content were immediately frozen on dry ice (−78 °C) and stored at −80 °C until analysis. Whole blood was allowed to clot overnight at 4 °C to release serotonin from platelet stores. Serum was separated by centrifugation at 12,000 rpm for 15 minutes at 4 °C and stored at −80 °C until analysis. Blood serotonin was determined in duplicate using a commercial radioimmunoassay Fast Track kit according to the manufacturer’s instructions (BA R-8900; Rocky Mountain Diagnostics, Colorado Springs, CO). Blood tryptophan levels were assessed in duplicate using a commercial ELISA kit according to the manufacturer’s instructions (BA E-2700; Rocky Mountain Diagnostics, Colorado Springs, CO).

### Mammary gland and duodenum serotonin extraction

Thawed mammary gland and duodenal tissues were washed twice in 200 μL ice cold 0.01 M phosphate buffered saline (PBS) to remove any excess blood, and then PBS was aspirated to remove excess liquid. Each tissue was placed into a clean glass homogenizer and 200 μL of ice cold 0.2 N perchloric acid was added. Tissues were homogenized using a Glas-Col tissue homogenizer (Cole-Parmer, Vernon Hills, IL). The tissue was fully homogenized by making eight complete iterations (pushing the pestle down, then bringing it back up). The homogenate was transferred to a clean microcentrifuge tube and then spun for 10 minutes at 2,000 x g at 4 °C. The middle layer of the supernatant from mammary gland homogenate was removed as the upper layer contained significant amounts of fat. The entire supernatant of the duodenal homogenate was removed. For determination of serotonin content via RIA (Rocky Mountain Diagnostics, Colorado Springs, CO), 25 μL of each supernatant was used. The remaining pellet was assayed for the quantitation of DNA via fluorometric assay according to the manufacturer’s instructions (Thermo Scientific/Pierce Protein Biology Products, Rockford, IL). All serotonin measurements from mammary gland and duodenal homogenates were normalized for the amount of DNA contained within each sample.

### Mammary gland histological evaluation

The right 4^th^ mammary gland was removed on d10 of the second lactation and fixed in 4% paraformaldehyde overnight at 4 °C. It was then transferred to 70% ethanol until dehydration with xylene and paraffin embedding. Paraffin blocks were sectioned to a thickness of 5 µm. For histological visualization, sectioned mammary glands were stained with hematoxylin and eosin (H&E) and imaged at 20x magnification. Five control, four *WAP-Cre* × *Tph1* ^FL/FL^, and four *WAP-Cre* × *Lrp5* ^FL/FL^ mammary gland sections were quantified for alveolar number and diameter using ImageJ software (NIH Version 1.49). Only alveoli whose borders were completely within the field of the image were counted and measured.

### Statistical Analysis

All data were analyzed using Prism Graph Pad (version 6.0). Serum serotonin and tryptophan, mammary gland and duodenal serotonin, and alveolar number and diameter were analyzed using a one-way analysis of variance (ANOVA) followed by Tukey’s multiple comparisons post hoc test for comparison of the means. Milk yield and pup growth were analyzed using a two-way ANOVA to examine the effects of genotype, time, and the interaction followed by Tukey’s multiple comparison post hoc test for comparison of the means. All data are presented as the mean ± standard error of the mean (SEM). Means were considered significantly different when *P* < 0.05.

### Data Availability

The datasets generated during and/or analyzed during the current study are available from the corresponding author on reasonable request.
